# Comparison of the Effects of Bipolar Membrane Preparation Conditions on the Mechanical Durability and Electrochemical Performance for Electrodialysis Applications

**DOI:** 10.1021/acsapm.5c03448

**Published:** 2026-07-08

**Authors:** Allison M. Crow, Julia D. Lenef, Todd G. Deutsch, Wilson A. Smith

**Affiliations:** † Department of Chemical and Biological Engineering, 1877 University of Colorado Boulder, Boulder, Colorado 80303, United States; ‡ Renewable and Sustainable Energy Institute, University of Colorado Boulder, Boulder, Colorado 80303, United States; § 53405 National Laboratory of the Rockies, Golden, Colorado 80401, United States

**Keywords:** electrodialysis, T-peel, bipolar membrane, adhesion, mechanical durability

## Abstract

Bipolar membranes (BPMs) are enabling materials for electrochemical conversion technologies such as water electrolysis, fuel cells, CO_2_ electrolysis, and electrodialysis (ED) for direct air/ocean capture of CO_2_. However, current BPM durability can suffer from chemical, mechanical, and performance degradation when operated at high current density (ion flux) and physical scale. Therefore, this limits its adoption in a wider applications space. BPMs have several known degradation mechanisms, including chemical breakdown of ion-exchange polymers, loss of junction adhesion, or physical breakdown due to shearing force and pressure swings in an electrodialysis cell. To assess the electrochemical stability and mechanical durability of BPMs under operational conditions, we investigated how fabrication conditions (including preconditioning, hot-pressing temperature and pressure, and catalyst loading) impact the adhesion of custom-made BPMs. T-peel studies were performed ex situ to quantify adhesive forces of BPMs, and bipolar membrane electrodialysis (BPMED) experiments were performed to assess the electrochemical performance of the corresponding BPMs. The results of this systematic comparison indicate that hydration and heated pressing create improved adhesion during the fabrication of BPMs, and BPMED testing shows that these fabrication techniques are not detrimental to the electrochemical performance of the BPMs.

## Graphical abstract

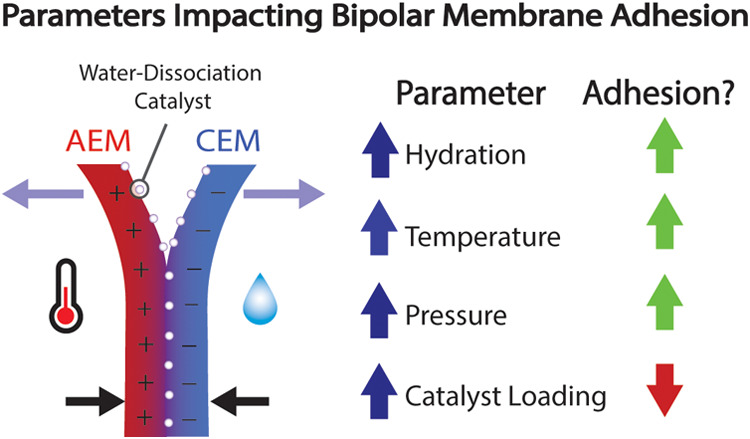

## Introduction

Ion-exchange membranes (IEMs) are permselective barriers that allow charge-compatible ions and water to move between areas of different chemical potential while preventing other ionic electrolyte components from crossing over. In electrochemical applications, IEMs are used to transport ions to balance the charge in a reactor when a voltage is applied between two end electrodes. Positively charged IEMs, or anion-exchange membranes (AEMs), are made up of compressed polymer chains that have fixed, positively charged functional groups that selectively allow the transport of anions while repelling cations. Conversely, negatively charged IEMs allow the transfer of cations while blocking anions, and are referred to as cation-exchange membranes (CEMs).^1^ A bipolar membrane (BPM) is a unique IEM that is made by the physical combination of an AEM and a CEM. BPMs combine anion- and cation-exchange membranes to separate liquid electrolytes and create or maintain a pH gradient between chambers in an electrochemical cell.^2–5^ In electrochemical systems, BPMs can be operated in forward or reverse bias based on whether the IEMs are oriented with the membrane charge matching that of the electrodes leading to water formation at the junction between the AEM and CEM (forward bias) or oriented opposite the electrode charge promoting water dissociation (reverse bias). When run in reverse bias, BPMs produce protons and hydroxides through water dissociation at the internal membrane junction. High-performance BPMs use a specific water-dissociation catalyst at the AEM/CEM interface to increase water-dissociation kinetics, and recent work has focused on tailoring the physical, chemical and electronic properties of water-dissociation catalysts.^6–11^

BPMs operated in reverse bias have been explored extensively for use in electrochemical systems including water electrolysis,^12,13^ fuel cells,^14–16^ CO_2_ reduction (CO2R),^17–19^ and electrodialysis, herein referred to as bipolar membrane electrodialysis (BPMED).^20–25^ In membrane electrode assembly (MEA) style reactors with only one anodic and one cathodic flow region, such as those found in water electrolysis, fuel cells, and CO2R, BPMs are desirable due to their ability to prevent co-ion leakage with less interest in the BPMs ability to generate protons and hydroxides. Conversely, in BPMED the key design component is the ability to generate acid and base to separate electrolyte chambers. Due to the differences in the end-use cases, BPMs also have unique design considerations based on their applications. In MEA or zero-gap reactors, the BPM is exposed to catalytic electrode reactions. Some AEM polymers degrade when exposed to the anodic oxidation^26^ reaction limiting the durability of the BPMs in these systems. Another consideration in zero-gap MEAs is the water available to maintain hydration of the BPM. In BPMED both of these concerns are alleviated due to the multicomponent design of the system. A BPMED reactor has multiple electrolyte compartments to isolate the BPM from the electrode reactions. Two main electrolyte compartments are positioned directly next to the BPM and are isolated from the electrolyte flow fields along the electrodes by monopolar membranes. This allows the BPM to be isolated for characterization without being affected by gas forming electrode reactions. BPMED systems are also characteristically liquid-flowing reactors, thus alleviating concerns about BPM dehydration during operation. These design characteristics of BPMED not only have industrial applications but also make BPMED an ideal platform for testing BPM performance isolated from competing degradation mechanisms.

In industry, BPMs have been largely utilized for electrodialysis applications such as wastewater treatment and brine remediation.^27–29^ Typically, in electrodialysis, BPMs are oriented in reverse bias to undergo water dissociation at the internal junction and supply protons and hydroxides to the dialysis cell. The acidification and basification of the solutions flowing adjacent to the BPM are used to convert salts, such as NaCl to NaOH and HCl, which can be concentrated, recovered, and utilized in downstream processes.^21,24,25^ The generation of acid and base can also be used to recover valuable chemicals from waste streams. Phosphate and ammonia can be recovered from biological waste, producing phosphoric acid and ammonium hydroxide,^22,28,30^ respectively, whereas lithium^31^ and boron^32^ can be recovered from environmental waste streams through electrochemically driven basification.^33^ BPMED systems are also used for a wide variety of inorganic and organic synthetic processes including the purification of several acids.^34,35^

Utilizing a similar pH-driven separations mechanism, BPMED is of increasing interest for integration with liquid alkaline direct air capture (DAC) processes to separate captured CO_2_ from the DAC effluent, which is typically a mixture of bicarbonate and carbonate, while regenerating the alkaline capture solvent for cyclic use. Initial assessments suggest that combining liquid alkaline DAC with a BPMED system for CO_2_ concentration/separation and solvent regeneration can lower overall net emissions compared to thermal CO_2_ regeneration, but high electricity costs currently challenge the feasibility of deploying this technology at large scale.^36^ In a BPMED system coupled with a direct air capture process, the acidification from protons delivered through the CEM side of the BPM releases CO_2_ from carbonate while hydroxides transport through the AEM side of the BPM to reconcentrate hydroxide ions in the alkaline electrolyte that is fed back to the air contactor.^20,37–41^ A similar approach may also be applicable for direct ocean capture and ocean alkalinity enhancement, where the carbonate feed would come from ocean water and undergo acidification and basification in the BPMED system.^42–46^ Despite their successful implementation in many industrial processes, BPMs still suffer from several limitations, including high energy demand and cost and low durability, which limit their deployment in energy applications.

In a BPMED reactor with free-standing membranes, i.e., not directly under compression from other cell components, adhesive forces between the individual IEMs are the primary mechanism keeping the BPM layers in contact. However, a common failure mechanism for BPMs is the loss of adhesion at the junction which decreases durability and energy efficiency of the BPMED device, which may lead to significant increases in the total operational costs. The interface between the AEM and CEM needs to be physically, chemically, and electronically robust in order to maintain high rates of water dissociation and ionic flux over long periods of time. To achieve this, an adhesive force that is composed of individual chemical, electrostatic, and physical forces is formed between the two monopolar membranes. Physical contact is necessary to minimize through-plane resistance, maintain high energy efficiency, and to create an electric field in which water dissociation occurs. To create durable, free-standing BPMs the properties that facilitate adhesion must be understood and optimized for the membranes and reactor conditions of choice.

Junction adhesion arises physically from van der Waals forces, physical entanglement of the polymer chains, and electrostatic attraction of fixed charges at the junction and the loss of these interactions result in failures of contact of the BPM junction by delamination, blistering, and rupture. Delamination occurs when there are minimal forces holding the monopolar membranes together before the membrane is subject to ionic flux forces. The complete lack of structural stability due to the fabrication makes these BPMs hard to handle prior to operation and unlikely to withstand electrochemically induced forces in operando, however the individual monopolar membranes may individually be unaffected. Blistering occurs when there is a buildup of gas, liquid, or solid species in the junction which forces the membranes to physically separate. Blisters can occur from gas formation, for example, if carbonate crosses into the junction and is then protonated to form CO_2_, from solids in the form of salt crystallization, or from liquid build up due to differential electrolyte or water flow to the junction. Rupture occurs when the in situ mechanical forces due to electrochemical operation acting on the BPM overcome forces promoting adhesion and pull the monopolar membranes away from one another. Ruptures may also result in damage to the monopolar membranes if the failure causes tears which could compromise the liquid–liquid separation in a flow cell.

The extent to which adhesion is created is due to the intrinsic properties of the individual IEM polymer chemistries including backbone structure, functional group, ion-exchange capacity (IEC), density, molecular weight, degree of cross-linking, and thickness. Properties such as IEC and the functional groups describe the extent of the fixed charge interactions that make up the electrostatics and van der Waals forces. While other bulk properties of the IEMs dictate the rate of transport through the polymers that characterize osmotic and Maxwell forces acting on the bulk structures. The pairing of the two IEM intrinsic properties in the formation of the BPM can also increase adhesive forces between the monopolar membranes. For example, Kao et al. showed higher adhesion between IEMs with the same backbone chemistries compared to those with nonsimilar backbones.^47^ A durable and energy-efficient BPM requires optimization of these material properties to manage trade-offs between resistivity, ion crossover, and physical swelling. For example, high ionic conductivity (high IEC) is good for achieving low through-plane resistance but may lead to junction rupture due to the increase in water uptake causing swelling. Balancing these opposing properties for membranes has been the focus of extensive research that has produced many new polymer compositions^4,48,49^ while a deeper understanding of the balance of forces has allowed BPM fabrication to be tailored to solve durability problems in electrochemical systems under relevant operational conditions.

There is a clear need to maintain junction adhesion in BPMs in order to achieve membranes suitable for large-scale, high-ion-flux applications. To help improve adhesion in BPMs, fabrication techniques involving changes in temperature, pressure, and solvent treatments have been used. Table 1 shows BPM fabrication conditions and membrane characteristics in a selection of recent BPMs fabrication methods shown in literature. Despite the variations in treatment processes, all of the literature shows the operation of BPMs with various degrees of efficiency in reverse bias, however, many suffer from poor stability in handling and low durability in operation. In the large-scale production of BPMs not only must the properties that determine adhesion be understood and controlled, but also the fabrication methods that impart these properties on the material should be considered and suitable for large-scale production. Membranes are typically manufactured on roll-to-roll lines in which hot pressing is done in a short, continuous processing “calendaring” event as part of an integrated process in which there is one line speed.^50–52^ As such, fabrication of membranes needs to be done in a relatively short time and in a way that integrates with membrane fabrication techniques. By understanding the most sensitive variables affecting adhesion we can decide how to optimize the large-scale production of durable BPMs. Despite the number of BPMs that have been made, no systematic study of how pretreatments and fabrication methods affect the adhesion strength and electrochemical performance of BPMs has been performed. In this paper, we isolate the effects of temperature, pressure, and hydration during the fabrication of BPMs from monopolar membranes for use in electrodialysis. We find that hot pressing at relatively higher temperatures improves mechanical stability of the BPM junction, while also providing lower cell voltages and higher energy efficiencies for BPMED.

**Table 1:** Fabrication Conditions for the Synthesis of Recently Reported BPMs Used in Reverse Bias Operations^a^,^b^

publication	membranes (AEM|CEM)	temperature	pressure	solvent	time
Kao et al. 2024.^47^	PiperION|Nafion	Unspecified	“no force”	None	None
	SEBS_AEL | SEBS_CEL				
Lucas et al. 2023.^53^	PiperION | Nafion	Ambient	“firmly between gloved fingers”	None	Unspecified
Al-Dhubhani et al. 2023.^54^	FAA-3 | SPEEK	150 °C	20 MPa	None	60 min
Powers et al. 2022.^55^	QPPO | SPEEK	121 °C	40,000 lbs^a^	3D -	15 min
				CEM – 15 min 1:1 DMAc:MeOH	
				AEM – 15 min 1:3 DMAc:MeOH	
Kole et al. 2021.^47^	QAPSf | SPEEK	120 °C	5000 lb	3D - Saturated 2-butanone (saturated acetone for perfluorinated BPMs) and dry nitrogen	30 min prior to solvent anneal
	Orion | SPEEK				1 h after solvent anneal
	PF AEM | Nafion				
Chen et al. 2020.^56^	PFAEM | Nafion	60 °C	3.38 MPa	2D – no	2 min
				3D – 15 min IPA	
Hohenadel et al. 2019.^57^	SPPB | HMT-PMBI	120 °C	10,000 lbs^a^	2D – 15 min	40 min
				3D – 15 min MeOH	
Shen et al. 2017.^58^	QPPO | SPEEK	121 °C	140 MPa	3D – 15 min DMF per side	30 min

a 3D membranes refer to membranes that have an electrospun fiber or lithographic patterned junction whereas 2D membranes are made from planar IEMs.

b Paper did not report area of applied force to calculate pressure.

## Methods

The AEM for the BPM fabrication, Aemion+, (with a thickness of 40 μm) was purchased from Ionomr and the CEM for BPM fabrication and for standalone CEM use in BPMED tests, Fumasep FKS (Fumatech GmbH, Germany), (with thickness of 30 μm) was purchased from Fuel Cell Store (Figure 1). Versogen (Versogen) (with thickness of 40 μm) was purchased from Versogen and used as the stand alone AEM in BPMED testing. HPLC grade (99.8%) isopropanol alcohol (IPA) was purchased from Sigma-Aldrich. TiO_2_–P25 (Nippon Aerosil Co., Ltd.) was suspended in a 50:50 IPA: deionized (DI) water (MilliQ 18 MΩ-cm). 1 M sodium hydroxide (NaOH), used as the basic electrolyte was made by dissolving NaOH pellets (Certified ACS, from Fisher Chemical) in DI water, and 1 M H_2_SO_4_, used as the acidic electrolyte, was made by diluting 5 N H_2_SO_4_ (Certified ACS Plus, from Fisher Chemical) with DI water.

**Figure 1:**
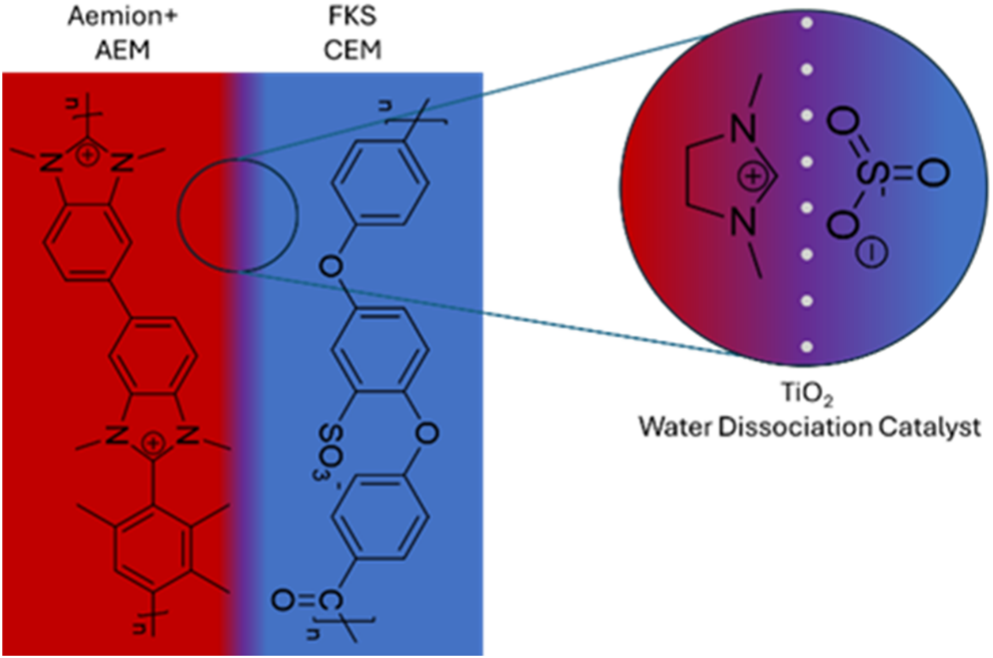
Anion and cation exchange membrane chemistries shown in a junction with TiO_2_ water dissociation catalyst. Opposite charged functional groups in BPMs help create adhesion and drive water dissociation.

### Fabrication of BPMs for T-peel experiments

AEMs and CEMs were cut to 4 cm × 2 cm pieces and the top 1.5 cm of the 4 cm length was covered with tape to facilitate T-peel testing. Taped membranes were then placed in their respective presoak conditions for >24 h. “Wet” membranes were soaked in DI water for at least 24 h and “dry” membranes were not presoaked.

### T-peel Testing

A T-peel test is a mechanical strength test used to assess adhesion between two flexible materials bonded together. A T-peel test was performed by pulling the two monopolar sides of a BPM apart at a 90 deg angle forming a “T” shape where the BPM is separating. T-peel adhesion data was measured with an Instron 5900 Series (Instron) using a 10 N load cells and pneumatic clamps. The taped sections of the prepared, wet BPM samples were placed in the clamps and pulled at 10 mm/min for 50 mm. The force needed to separate the materials over time was measured as a force curve, shown in Figure 2a. Force per meter adhesion data was processed by averaging force data from 40 to 110 s and dividing that by the width of the pull samples (20 mm).

**Figure 2:**
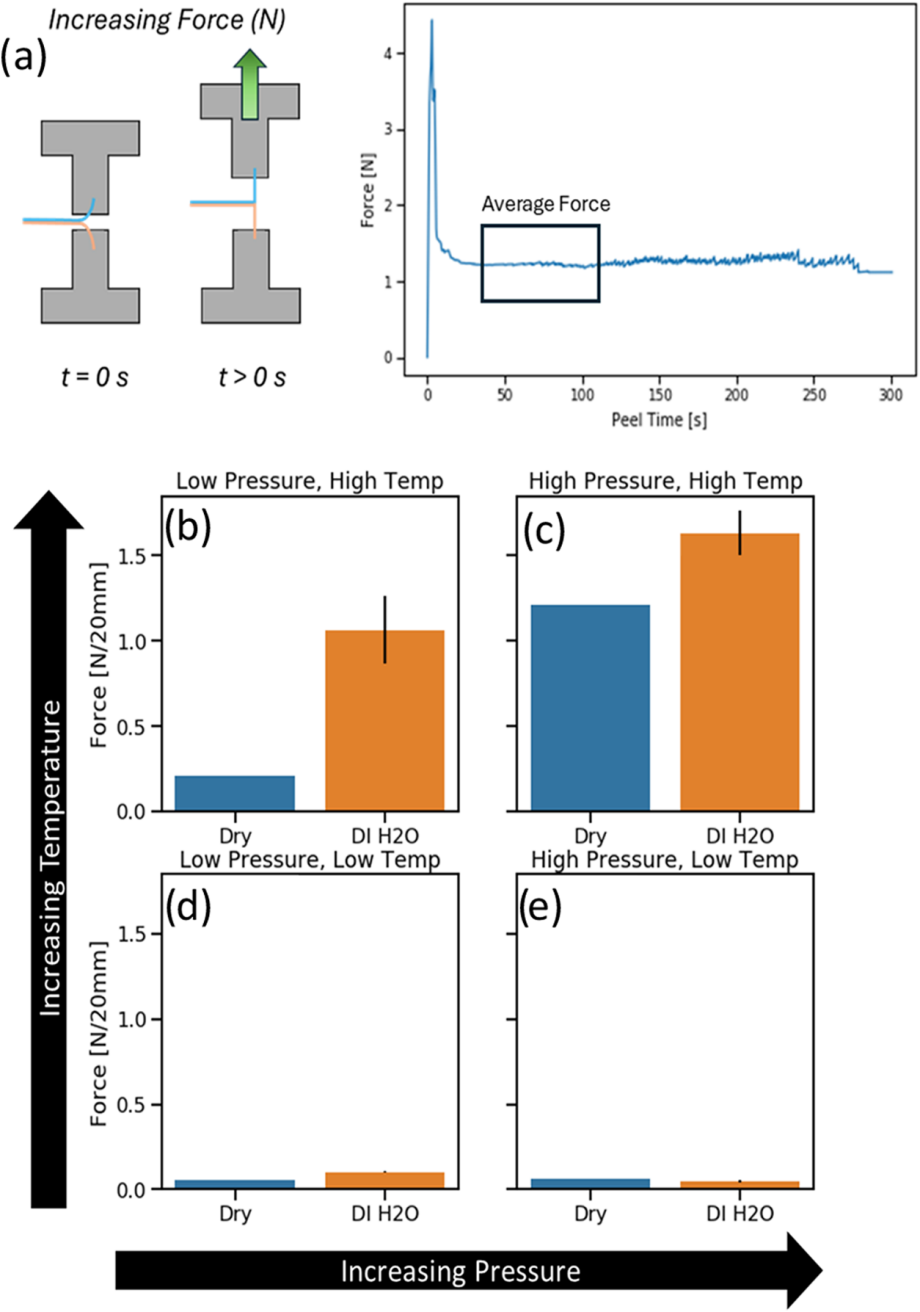
Adhesion forces measured by T-peeling BPMs fabricated under different presoak and hot press conditions. T-peel tests measure peel separation force of two flexible materials that have been bonded together. The force is calculated from an average of the force overtime applied to peel the membranes apart (a). Temperatures were 25 °C (d, e) and 100 °C (b, c). Pressures were 0.14 MPa (b, d) and 0.69 MPa (c, e).

To adhere the AEM and CEM to make a BPM for T-peel testing, the AEM was placed on a 4 cm × 4 cm piece of Garlock Gylon 3540 (1/16 in. thick) covered with Kapton tape to prevent sticking. The CEM was placed on top of the AEM followed by another piece of Kapton tape and 4 cm × 4 cm Gylon. The two membranes were then placed between two metal plates in a hot press (Carver) set at 25 or 100 °C. The membranes were pressed for two minutes and then removed and placed either between wet paper towels or in fresh DI water. All BPMs were hydrated after fabrication and hydration was maintained through all testing.

For catalyzed T-peel BPM samples, low, medium, and high catalyst loadings were made by serial dilution of a stock 2 mg/mL TiO_2_ in 50:50 IPA:DI solution air sprayed on one monopolar membrane before combining into a BPM. Air spraying is a catalyst dispersion technique that uses compressed gas, in this case nitrogen, to uniformly disperse the nanoparticle water dissociation catalyst solution onto the membrane surface. The catalyst was suspended by sonication for 30 min in a room-temperature, water-bath sonicator. Soaked membranes, typically the AEM, except in the test where spraying on the AEM versus the CEM was compared, were air sprayed with TiO_2_ catalyst suspended in 50:50 IPA:DI solution using an air sprayer Master Airbrush Model E91, 0.8 mm tip, (Amazon) connected to a house nitrogen stream. After spraying, the membranes were sandwiched and pressed in the same process as the uncatalyzed membranes. A summary of the test matrix used is shown in Table 2.

**Table 2:** Fabrication Conditions for BPMs were Studied for Their Effect on the Junction Adhesion

temperature	pressure	presoak (>24 h)
25 °C	0.139 MPa	None
		DI Water
	0.695 MPa	None
		DI Water
100 °C	0.139 MPa	None
		DI Water
	0.695 MPa	None
		DI Water

### Bipolar Membrane Electrodialysis (BPMED) Testing

BPMs with and without water-dissociation catalysts were prepared for BPMED testing following the same procedures as the samples prepared for T-peel testing, except that the membranes were 3.81 cm × 3.81 cm. Membranes were stored at least 24 h in DI water after pressing to allow them to equilibrate before electrochemical testing.

The electrochemical performance of these BPMs was evaluated in a custom-designed BPMED cell (Figure 3a). The cell was designed to assess electrochemical properties of BPMs by placing reference electrodes near the BPM while maintaining flowing electrolytes. The flow field used had a 4 cm^2^ square active area. Platinum foil was used for the anode and cathode to serve as the catalyst for the oxygen evolution reaction (OER) and hydrogen evolution reaction (HER), respectively. A 30-μm-thick Fumasep FKS CEM separates flow chambers on the anodic side and a 40-μm-thick Versogen AEM separates flow chambers on the cathodic side of the BPM.

**Figure 3:**
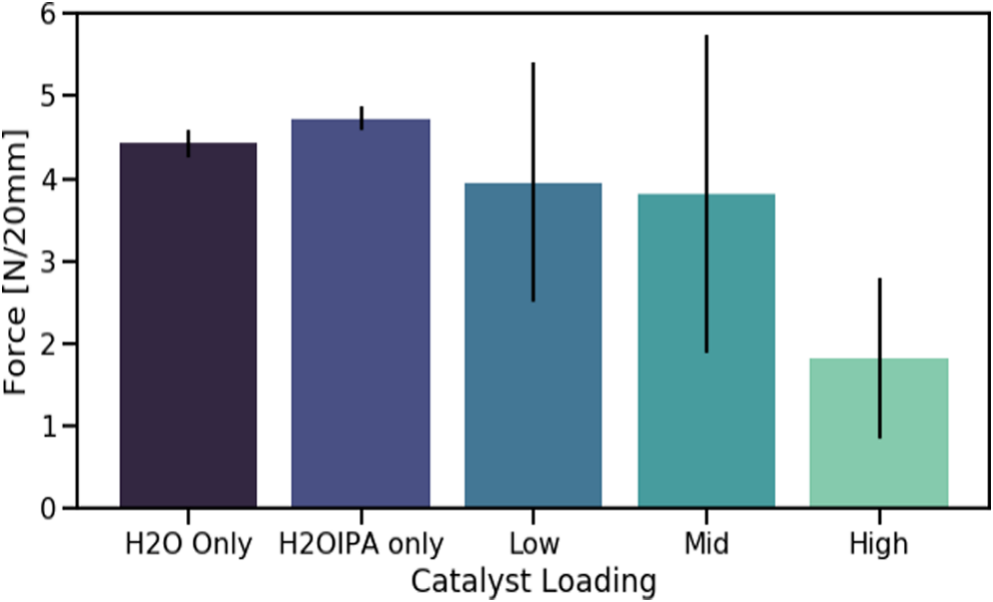
Adhesion force required to pull apart BPMs made with different catalyst loadings. Catalyst loadings were made through serial dilution of a concentrated solution.

The electrochemical performance of the BPMs was examined using 1 M NaOH in the anodic chambers and 1 M H_2_SO_4_ in the cathodic electrolyte flow chambers flowing at 30 sccm and at room temperature. 500 mL of electrolyte recirculated per chamber. A Koslow Hg/HgO reference electrode (0.1 V vs NHE) was used in the base chamber, and a Koslow Hg/HgSO_4_ reference electrode (0.64 V vs NHE) was used in the acid chamber. To assess the electrochemical performance of the BPMs, a galvanodynamic program was run, and data was collected between reference electrodes in a 4-electrode configuration, isolating the voltage–current responses of the BPM. Data was collected from 10 to 125 mA/cm^2^at 25 mA/s. Transmembrane voltage data was corrected by +0.54 V to account for the different reference potentials from the dissimilar reference electrodes.

### Materials Characterization

Scanning electron microscopy (SEM) and energy dispersive X-ray spectroscopy (EDS) were performed using a ThermoFisher Nova SEM 630. A beam voltage of 10 kV and current of 2.6 nA were selected for both SEM imaging and EDS chemical mapping. Approximately 5 nm of Pt was sputtered on T-peeled surfaces to minimize charging.

## Results and Discussion

### Effects of BPM Fabrication Conditions on Junction Adhesion

T-peel measurements were obtained for BPMs made under different fabrication conditions to measure the force required to separate the individual monopolar membranes, summarized in Figure 2. High adhesion is correlated with a high T-peel force measurement. Two different preparation methods were compared at four different hot-press conditions.

Membranes hydrated in DI water prior to hot pressing showed higher adhesion than dry membranes, while membranes pressed at a 100 °C (Figure 2b,c) show strong adhesion compared to those pressed at 25 °C (Figure 2d,e). While higher temperatures were needed to create a measurable T-peel force, pressing the BPMs with higher pressure further increased the adhesion for both dry and wet prepared membranes.

### Catalyzing the BPM Junction Creates Lower and More Variable Adhesion Forces

BPMs without a water dissociation catalyst will require much higher potential to drive water dissociation at the junction, thus, it is important to understand the effects of catalyst loading on adhesion. Utilizing the highest adhesion presoak and hot-press conditions (water hydrated, high temperature, high pressure) membranes were prepared with increasing catalyst loadings.

To introduce the water-dissociation catalyst to the junction of the AEM and CEM, suspended P25 TiO_2_ catalyst was air sprayed on to one membrane then pressed into the second monopolar membrane. To deconvolute the adhesion results that may derive from the air spraying procedure or from the IPA and water suspension solution the catalysts were suspended and sprayed in, two controls were considered – spraying just DI water and spraying 50:50 IPA:DI water. The addition of IPA slightly increased the adhesion force measured compared to just spraying water. Figure 3 shows that the addition of the water-dissociation catalyst lowers the BPM junction adhesion force and that the addition of higher loadings of catalyst tends to lower the adhesion. The catalyzed T-peel samples show large standard deviations which can be attributed to the nonuniformity of the junction created by introducing the water-dissociation catalyst between the adhesive layers. Figure S1 shows the comparison of the force curves between catalyzed and uncatalyzed membranes. The noncatalyzed samples produce flat force curve profiles over time while the catalyzed samples show fluctuations in force measured along the length of the test sample.

In addition to observing the effect of catalyst loading on adhesive force, the effects of spraying the catalyst on different parts of the BPM were also evaluated. When the catalyst was sprayed on the AEM and then pressed to the CEM, the T-peel results showed higher adhesion at every catalyst loading compared to when the catalyst was sprayed on the CEM (Figure S2). The application of IPA to the AEM may partially solvate a surface layer of the material, promoting adhesion when pressed to the CEM.

To further understand the effects of the catalyst on the junction adhesion, scanning electron spectroscopy (SEM) - energy-dispersive X-ray spectroscopy (EDS) was used to image catalyzed T-peel samples after the peel test was completed. Figure 4 shows the SEM-EDS imaging of a TiO_2_ catalyzed BPM after T-peeling, which had previously demonstrated high-adhesion with a medium catalyst loading. Titanium from the water-dissociation catalyst can be observed on both membrane surfaces but is dominant on the CEM (Figure 4a–d). The catalyst was only sprayed on to the AEM which suggests that the mechanism of failure is dictated by the interparticle catalyst interactions and not between the monopolar membranes and the catalyst particles. This is expected since TiO_2_ interparticle attraction is several orders of magnitude smaller than forces measured between membranes^59^ and further confirms the decrease in adhesion force measured with increasing catalyst loading.

**Figure 4:**
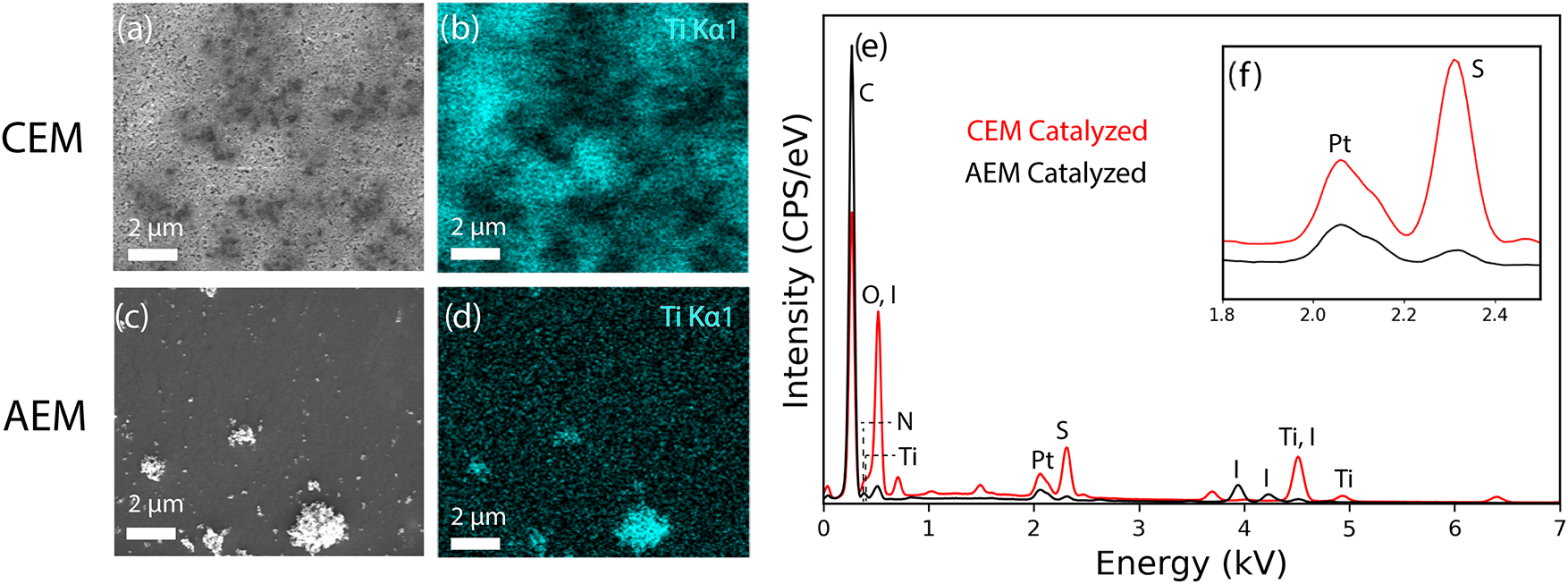
Scanning electron microscopy (SEM) and energy-dispersive X-ray spectroscopy (EDS) of the medium loading TiO_2_ catalyzed BPM (a) SEM image of the CEM (b) corresponding Ti EDS map (c) SEM image of the AEM (d) corresponding Ti EDS (e) EDS spectra of the CEM and AEM from regions (a) and (c) and (f) inset zoom of the S from the CEM and AEM. Five nm of Pt was sputtered on the surface to minimize charging and was therefore present in the EDS spectra.

There is also evidence of sulfur on the AEM in the EDS spectra (Figure 4e,f), indicating that there may be a minor contribution of mechanical failure from the monopolar membrane interactions. Comparing the uncatalyzed sample with identical hot-pressing conditions, the membranes that showed high adhesion forces also show a textured shearing pattern characteristic of membranes being pulled indicating that the membranes are well adhered when the peel force is applied (Figure S3). In these shearing patterns, sulfur from the CEM polymers, can be seen on the AEM, indicating that the CEM and AEM adhere well to each other, further supporting the mechanical weakness of BPMs is partially due to the lack of sufficient interfacial surface area contact between the monopolar membranes. This effect is also present in the low pressure, high temperature sample (Figure S4), with the presence of sulfur on the AEM. Finally, to confirm the presence of sulfur in these cases, EDS spectra of the control CEM and AEM were obtained, and no sulfur was present in the starting monopolar AEM (Figure S5). Previous work has sought to balance high adhesion with sufficient water dissociation catalyst loadings through either patterned catalyst application or electrospun junctions.^54–56,60–62^ Both fabrication techniques seek to increase surface contact between the monopolar membranes in a bipolar membrane junction to improve junction durability and electrochemical performance.

### Relating Ex Situ Adhesion to In Situ BPMED Resistance

To understand if the ex-situ T-peel characterization reveals any correlative trends relevant to the electrochemical performance, BPMs were prepared for testing in a 4 cm^2^ active area BPMED system. For these tests, all membranes were pretreated in a >24-h soak in DI water. Membranes pressed under different pressures and temperatures, and with and without water-dissociation catalysts were compared using a BPMED cell connected to a SP-300 Biologic potentiostat to assess voltage responses across the BPM. Figure 5b shows the polarization curves collected across the BPM for the fabrication conditions tested. TiO_2_-catalyzed BPMs exhibit lower voltages as the current density is increased compared to uncatalyzed BPMs, as expected. For both catalyzed and uncatalyzed BPMs, the same trend is apparent between fabrication conditions and lower operational voltage; high-temperature pressing lowers the voltage measured across the BPM.

**Figure 5:**
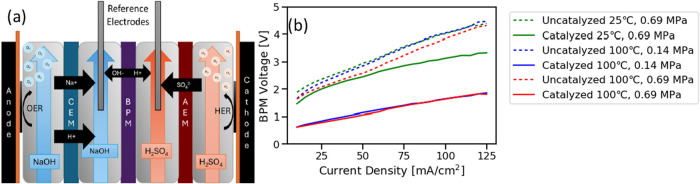
Side profile of custom-designed sensing BPMED cell with a four-electrode set up allowing direct electrochemical measurements isolating the bipolar membrane in a flowing reactor (a). Polarization curves collected across BPMs with different fabrication conditions. Dashed lines indicate noncatalyzed BPMs while solid lines indicate those catalyzed with TiO_2_. BPMs that had higher adhesion force values in T-peel testing show lower resistance as indicated by the lowered BPM voltage at the same current density (b).

The pretreatment and fabrication procedures used to make BPMs from commercially available AEM, CEM, and metal-oxide water-dissociation catalysts change the ex-situ measured adhesion force and the in situ measured voltages at different applied current densities. Looking at the forces acting on a BPM, strategies for improving adhesion during fabrication, particularly those compatible with scaled production, can be developed.

Hydration of the AEM and CEM prior to assembly expands membranes to their wet thickness (∼10 μm) which is similar to the hydration conditions the membranes experience during electrodialysis. By adhering the membranes after they have swollen from hydration, sheer forces created by the two membranes’ differential swelling can be minimized, preventing failure due to rupture at the junction *in situ*.

As shown in Table 1, using a hot press to increase adhesion is a common practice for BPM fabrication, however, there has been little discussion in literature for the goals of utilizing temperature and pressure and their effects on electrochemical performance. A BPM differs from its individual monopolar components when a junction is formed in which water dissociation occurs under reverse bias. This junction requires physical contact between the AEM and CEM, or having both membranes adhere to the same nanoscale water-dissociation catalyst. When membranes are subject to heat sufficiently close to but under their glass-transition temperature, slight softening will occur. When hot pressing, softened membranes are forced together establishing more contact between membranes and physically entangles the membranes at the junction. This increased contact and increased fixed-charge interaction improves the attraction between the monopolar membranes. Solvent vapor exposure can also be used to similarly soften the monopolar membranes to improve adhesion.^60^

Adhesion between the membranes is complicated by the trade-off of improved BPM potential with the addition of a water-dissociation catalyst. In BPMED testing, catalyzed BPMs resulted in more than 2 V lower potential at 100 mA/cm^2^ for high-temperature-treated BPMs, however, the introduction of a TiO_2_ catalyst lowers the force between the membranes measured by T-peel, regardless of loading, which may indicate lower durability of those junctions. We acknowledge that the *ex situ* adhesion measurements are not a direct reflection of the mechanical stability of the BPMs under relevant electrochemical conditions. This reduction in *ex situ* adhesion may be purely due to the decrease in surface contact between membranes caused by the presence of nanoparticles but may also be due to a disruption in fixed charge interactions. The challenge of designing a BPM interface for high performance versus high durability may be able to be addressed through the fabrication of 3D electrospun junctions that can have remarkably high interfacial surface area between membranes. However, the establishment of a 3D junction may also change the water dissociation catalyst microenvironment and species transport in the junction. Further work is needed to explore 3D fabrication strategies to improve BPM adhesion and electrochemical performance.

## Conclusions

In this work, we show that BPM processing conditions such as temperature, pressure and hydration affect both BPM ex-situ adhesion and in situ electrochemical performance during electrodialysis. We show that ex-situ T-peel testing is one way to study how material and fabrication conditions affect the adhesion of a BPM internal junction. From the T-peel testing, it was found that prehydrating membranes helps minimize forces counter to the junction adhesion. It was also shown that pressing at elevated temperatures is necessary for the preparation of a well-adhered junction, and hot pressing at moderate pressure further improved adhesion. Through testing in a BPMED cell, it was shown that an increase in temperature to the preparation method did not negatively affect the voltage of these BPMs and may actually improve the BPM performance during electrodialysis.

It was also shown that the addition of a water-dissociation catalyst to the BPM junction lowers the junction adhesion but also lowers the potential across the BPM. This trade off must be carefully balanced to maintain high efficiency and long lifetime performance for electrodialysis. The creation of high surface area, 3D BPMs may help alleviate this competition by increasing AEM and CEM surface contact while maintaining sufficient catalyst loading to promote high- rate water dissociation.

To improve BPM durability and create scalable manufacturing processes, future work should evaluate solvent vapor effects by connecting polymer chemistry with compatible solvents and temperatures for softening. It will also be necessary to explore how these pretreatment steps can be done on roll-to-roll manufacturing lines. In addition to solvent, the exact time for each processing step can be further refined and connected to intrinsic polymer characteristics. The list of variables that may impact the durability of BPM fabrication is extensive, and durability testing *in situ* is time intensive. Connecting T-peel adhesion force values with long-term adhesion *in operando* will allow rapid development of durable, high-performance BPMs.

## Supplementary Material



## Notes

Additional forces curves and SEM details (PDF)

## References

[1] Jiang S., Sun H., Wang H., Ladewig B. P., Yao Z. (2021). A Comprehensive Review
on the Synthesis and Applications of Ion Exchange Membranes. Chemosphere.

[2] Pärnamäe R., Mareev S., Nikonenko V., Melnikov S., Sheldeshov N., Zabolotskii V., Hamelers H. V. M., Tedesco M. (2021). Bipolar Membranes:
A Review on Principles, Latest Developments, and Applications. J. Membr. Sci..

[3] Giesbrecht P. K., Freund M. S. (2020). Recent Advances in Bipolar Membrane
Design and Applications. Chem. Mater..

[4] Blommaert M. A., Aili D., Tufa R. A., Li Q., Smith W. A., Vermaas D. A. (2021). Insights and Challenges for Applying Bipolar Membranes
in Advanced Electrochemical Energy Systems. ACS Energy Lett..

[5] Tufa R. A., Blommaert M. A., Chanda D., Li Q., Vermaas D. A., Aili D. (2021). Bipolar Membrane
and Interface Materials for Electrochemical Energy
Systems. ACS Appl. Energy Mater..

[6] Mareev S. A., Evdochenko E., Wessling M., Kozaderova O. A., Niftaliev S. I., Pismenskaya N. D., Nikonenko V. V. (2020). A Comprehensive
Mathematical Model of Water Splitting in Bipolar Membranes: Impact
of the Spatial Distribution of Fixed Charges and Catalyst at Bipolar
Junction. J. Membr. Sci..

[7] Chen L., Xu Q., Oener S. Z., Fabrizio K., Boettcher S. W. (2022). Design
Principles for Water Dissociation Catalysts in High-Performance Bipolar
Membranes. Nat. Commun..

[8] Yang C., Wu Z., Zhao Z., Gao Y., Ma T., He C., Wu C., Liu X., Luo X., Li S., Cheng C., Zhao C. (2023). Electronic Structure-Dependent
Water-Dissociation Pathways of Ruthenium-Based
Catalysts in Alkaline H2-Evolution. Small.

[9] Chen L., Xu Q., Boettcher S. W. (2023). Kinetics and Mechanism of Heterogeneous Voltage-Driven
Water-Dissociation Catalysis. Joule.

[10] Sasmal S., Chen L., Sarma P. V., Vulpin O. T., Simons C. R., Wells K. M., Spontak R. J., Boettcher S. W. (2024). Materials
Descriptors for Advanced Water Dissociation Catalysts in Bipolar Membranes. Nat. Mater..

[11] Bui J. C., Corpus K. R. M., Bell A. T., Weber A. Z. (2021). On the Nature of
Field-Enhanced Water Dissociation in Bipolar Membranes. J. Phys. Chem. C.

[12] Mayerhöfer B., McLaughlin D., Böhm T., Hegelheimer M., Seeberger D., Thiele S. (2020). Bipolar Membrane Electrode Assemblies
for Water Electrolysis. ACS Appl. Energy Mater..

[13] Tricker A. W., Lee J. K., Babbe F., Shin J. R., Weber A. Z., Peng X. (2023). Engineering Bipolar Interfaces for Water Electrolysis Using Earth-Abundant
Anodes. ACS Energy Lett..

[14] Peng S., Xu X., Lu S., Sui P.-C., Djilali N., Xiang Y. (2015). A Self-Humidifying
Acidic–Alkaline Bipolar Membrane Fuel Cell. J. Power Sources.

[15] Belhaj I., Faria M., Šljukić B., Geraldes V., Santos D. M. F. (2023). Bipolar Membranes for Direct Borohydride
Fuel Cells—A
Review. Membranes.

[16] Ahlfield J. M., Liu L., Kohl P. A. (2017). PEM/AEM
Junction Design for Bipolar Membrane Fuel Cells. J. Electrochem. Soc..

[17] Nature R. C. by S . Bipolar membrane electrolyzers enable high single-pass CO2 electroreduction. Research Communities by Springer Nature. http://chemistrycommunity.nature.com/posts/bipolar-membrane-electrolyzers-enable-high-single-pass-co2-electroreduction. (accessed 2024–08–25).

[18] Petrov K. V., Koopman C. I., Subramanian S., Koper M. T. M., Burdyny T., Vermaas D. A. (2024). Bipolar Membranes for Intrinsically Stable and Scalable
CO2 Electrolysis. Nat. Energy.

[19] Yan Z., Hitt J. L., Zeng Z., Hickner M. A., Mallouk T. E. (2021). Improving
the Efficiency of CO2 Electrolysis by Using a Bipolar Membrane with
a Weak-Acid Cation Exchange Layer. Nat. Chem..

[20] Iizuka A., Hashimoto K., Nagasawa H., Kumagai K., Yanagisawa Y., Yamasaki A. (2012). Carbon Dioxide Recovery from Carbonate Solutions Using
Bipolar Membrane Electrodialysis. Sep. Purif.
Technol..

[21] Chen T., Bi J., Ji Z., Yuan J., Zhao Y. (2022). Application of Bipolar
Membrane Electrodialysis for Simultaneous Recovery of High-Value Acid/Alkali
from Saline Wastewater: An in-Depth Review. Water Res..

[22] Li Y., Wang R., Shi S., Cao H., Yip N. Y., Lin S. (2021). Bipolar Membrane Electrodialysis
for Ammonia Recovery from Synthetic
Urine: Experiments, Modeling, and Performance Analysis. Environ. Sci. Technol..

[23] Kingsbury R. S., Chu K., Coronell O. (2015). Energy Storage
by Reversible Electrodialysis: The Concentration
Battery. J. Membr. Sci..

[24] Sun Y., Wang Y., Peng Z., Liu Y. (2022). Treatment of High Salinity
Sulfanilic Acid Wastewater by Bipolar Membrane Electrodialysis. Sep. Purif. Technol..

[25] Jiang G., Li H., Xu M., Ruan H. (2021). Sustainable
Reverse Osmosis, Electrodialysis
and Bipolar Membrane Electrodialysis Application for Cold-Rolling
Wastewater Treatment in the Steel Industry. J. Water Process Eng..

[26] Parrondo J., Wang Z., Jung M.-S. J., Ramani V. (2016). Reactive Oxygen
Species
Accelerate Degradation of Anion Exchange Membranes Based on Polyphenylene
Oxide in Alkaline Environments. Phys. Chem.
Chem. Phys..

[27] Yang Y., Gao X., Fan A., Fu L., Gao C. (2014). An Innovative Beneficial
Reuse of Seawater Concentrate Using Bipolar Membrane Electrodialysis. J. Membr. Sci..

[28] Lv Y., Yan H., Yang B., Wu C., Zhang X., Wang X. (2018). Bipolar Membrane
Electrodialysis for the Recycling of Ammonium Chloride Wastewater:
Membrane Selection and Process Optimization. Chem. Eng. Res. Des..

[29] Ilhan F., Kabuk H. A., Kurt U., Avsar Y., Gonullu M. T. (2016). Recovery
of Mixed Acid and Base from Wastewater with Bipolar Membrane Electrodialysis—a
Case Study. Desalination Water Treat..

[30] van
Linden N., Bandinu G. L., Vermaas D. A., Spanjers H., van Lier J. B. (2020). Bipolar Membrane Electrodialysis for Energetically
Competitive Ammonium Removal and Dissolved Ammonia Production. J. Clean. Prod..

[31] Meshram P., Pandey B. D., Mankhand T. R. (2014). Extraction of Lithium
from Primary
and Secondary Sources by Pre-Treatment, Leaching and Separation: A
Comprehensive Review. Hydrometallurgy.

[32] Nagasawa H., Iizuka A., Yamasaki A., Yanagisawa Y. (2011). Utilization
of Bipolar Membrane Electrodialysis for the Removal of Boron from
Aqueous Solution. Ind. Eng. Chem. Res..

[33] İpekçi D., Kabay N., Bunani S., Altıok E., Arda M., Yoshizuka K., Nishihama S. (2020). Application
of Heterogeneous Ion Exchange Membranes for Simultaneous Separation
and Recovery of Lithium and Boron from Aqueous Solution with Bipolar
Membrane Electrodialysis (EDBM). Desalination.

[34] Fu L., Gao X., Yang Y., Aiyong F., Hao H., Gao C. (2014). Preparation
of Succinic Acid Using Bipolar Membrane Electrodialysis. Sep. Purif. Technol..

[35] Hülber-Beyer É., Bélafi-Bakó K., Nemestóthy N. (2021). Low-Waste
Fermentation-Derived Organic Acid Production by Bipolar Membrane Electrodialysis—an
Overview. Chem. Pap..

[36] Küng L., Aeschlimann S., Charalambous C., McIlwaine F., Young J., Shannon N., Strassel K., Maesano C. N., Kahsar R., Pike D., van der Spek M., Garcia S. (2023). A Roadmap for Achieving Scalable,
Safe, and Low-Cost
Direct Air Carbon Capture and Storage. Energy
Environ. Sci..

[37] Ruan H., Wu S., Chen X., Zou J., Liao J., Cui H., Dong Y., Qiu Y., Shen J. (2022). Capturing CO2 with
NaOH Solution from Reject Brine via an Integrated Technology Based
on Bipolar Membrane Electrodialysis and Hollow Fiber Membrane Contactor. Chem. Eng. J..

[38] Eisaman M. D., Alvarado L., Larner D., Wang P., Garg B., Littau K. A. (2011). CO2 Separation Using Bipolar Membrane
Electrodialysis. Energy Environ. Sci..

[39] Ye W., Huang J., Lin J., Zhang X., Shen J., Luis P., Van der
Bruggen B. (2015). Environmental Evaluation of Bipolar
Membrane Electrodialysis for NaOH Production from Wastewater: Conditioning
NaOH as a CO2 Absorbent. Sep. Purif. Technol..

[40] Shu Q., Sin C. S., Tedesco M., Hamelers H. V. M., Kuntke P. (2023). Optimization
of an Electrochemical Direct Air Capture Process with Decreased CO2
Desorption Pressure and Addition of Background Electrolyte. Chem. Eng. J..

[41] Valluri S., Kawatra S. K. (2021). Reduced Reagent Regeneration Energy
for CO2 Capture
with Bipolar Membrane Electrodialysis. Fuel
Process. Technol..

[42] Digdaya I. A., Sullivan I., Lin M., Han L., Cheng W.-H., Atwater H. A., Xiang C. (2020). A Direct Coupled Electrochemical
System for Capture and Conversion of CO2 from Oceanwater. Nat. Commun..

[43] Sharifian R., van der Wal H. C., Wagterveld R. M., Vermaas D. A. (2023). Fouling Management
in Oceanic Carbon Capture via In-Situ Electrochemical Bipolar Membrane
Electrodialysis. Chem. Eng. J..

[44] Zhao Y., Wang J., Ji Z., Liu J., Guo X., Yuan J. (2020). A Novel Technology of Carbon Dioxide
Adsorption and Mineralization
via Seawater Decalcification by Bipolar Membrane Electrodialysis System
with a Crystallizer. Chem. Eng. J..

[45] Eisaman M. D., Parajuly K., Tuganov A., Eldershaw C., Chang N., Littau K. A. (2012). CO2 Extraction from
Seawater Using
Bipolar Membrane Electrodialysis. Energy Environ.
Sci..

[46] Sharifian R., Boer L., Wagterveld R. M., Vermaas D. A. (2022). Oceanic Carbon Capture
through Electrochemically Induced in Situ Carbonate Mineralization
Using Bipolar Membrane. Chem. Eng. J..

[47] Kao Y.-L., Chen L., Boettcher S. W., Aili D. (2024). Divergent Synthesis
of Bipolar Membranes Combining Strong Interfacial Adhesion and High-Rate
Capability. ACS Energy Lett..

[48] Bui J. C., Lees E. W., Marin D. H., Stovall T. N., Chen L., Kusoglu A., Nielander A. C., Jaramillo T. F., Boettcher S. W., Bell A. T., Weber A. Z. (2024). Multi-Scale
Physics
of Bipolar Membranes in Electrochemical Processes. Nat. Chem. Eng..

[49] Narayanaru S., Miyanishi S., Kuroki H., Anilkumar G. M., Yamaguchi T. (2023). Start–Stop
Cyclic Durability Analysis of Membrane–Electrode
Assemblies Using Polyflourene-Based Electrolytes for an Anion-Exchange
Membrane Water Electrolyzer. ACS Sustainable
Chem. Eng..

[50] Chen J., Liu H., Huang Y., Yin Z. (2016). High-Rate
Roll-to-Roll Stack and
Lamination of Multilayer Structured Membrane Electrode Assembly. J. Manuf. Process..

[51] Steenberg T., Hjuler H. A., Terkelsen C., Sánchez M. T. R., Cleemann L. N., Krebs F. C. (2012). Roll-to-Roll Coated
PBI Membranes
for High Temperature PEM Fuel Cells. Energy
Environ. Sci..

[52] Chen J., Jiang X., Tang W., Ma L., Li Y., Huang Y., Yin Z. (2020). Roll-to-Roll Stack and Lamination
of Gas Diffusion Layer in Multilayer Structured Membrane Electrode
Assembly. Proc. Inst. Mech. Eng. Part B J. Eng.
Manuf..

[53] Lucas É., Bui J., Hwang M., Wang K., Bell A., Weber A., Ardo S., Atwater H., Xiang C. (2023). Asymmetric
Bipolar Membrane for High Current Density Electrodialysis Operation
with Exceptional Stability. ACS Energy Lett..

[54] Al-Dhubhani E., Post J. W., Duisembiyev M., Tedesco M., Saakes M. (2023). Understanding
the Impact of the Three-Dimensional Junction Thickness of Electrospun
Bipolar Membranes on Electrochemical Performance. ACS Appl. Polym. Mater..

[55] Powers D., Mondal A. N., Yang Z., Wycisk R., Kreidler E., Pintauro P. N. (2022). Freestanding Bipolar
Membranes with an Electrospun
Junction for High Current Density Water Splitting. ACS Appl. Mater. Interfaces.

[56] Chen Y., Wrubel J. A., Klein W. E., Kabir S., Smith W. A., Neyerlin K. C., Deutsch T. G. (2020). High-Performance
Bipolar Membrane
Development for Improved Water Dissociation. ACS Appl. Polym. Mater..

[57] Hohenadel A., Powers D., Wycisk R., Adamski M., Pintauro P., Holdcroft S. (2019). Electrochemical
Characterization of Hydrocarbon Bipolar
Membranes with Varying Junction Morphology. ACS Appl. Energy Mater..

[58] Shen C., Wycisk R., Pintauro P. N. (2017). High Performance
Electrospun Bipolar
Membrane with a 3D Junction. Energy Environ.
Sci..

[59] Salameh S., Schneider J., Laube J., Alessandrini A., Facci P., Seo J. W., Ciacchi L. C., Mädler L. (2012). Adhesion Mechanisms
of the Contact Interface of TiO2 Nanoparticles in Films and Aggregates. Langmuir.

[60] Kole S., Venugopalan G., Bhattacharya D., Zhang L., Cheng J., Pivovar B., Arges C. G. (2021). Bipolar Membrane Polarization Behavior
with Systematically Varied Interfacial Areas in the Junction Region. J. Mater. Chem. A.

[61] Xu Z., Wan L., Liao Y., Pang M., Xu Q., Wang P., Wang B. (2023). Continuous
Ammonia Electrosynthesis Using Physically Interlocked
Bipolar Membrane at 1000 mA Cm–2. Nat.
Commun..

[62] Lu H., Wang L., Wycisk R., Pintauro P. N., Lin S. (2020). Quantifying
the Kinetics-Energetics Performance Tradeoff in Bipolar Membrane Electrodialysis. J. Membr. Sci..

