# Supplementary material for: Comparison of the Effects of Bipolar Membrane Preparation Conditions on the Mechanical Durability and Electrochemical Performance for Electrodialysis Applications
Source: ACS Appl Polym Mater. 2026 Jul 8;8(14):10959–68. doi: 10.1021/acsapm.5c03448 (PMC13410297; doi:10.1021/acsapm.5c03448)
Supplement: Supplementary file 1 [file ap5c03448_si_001.pdf]

Supporting Information

Comparison of the effects of bipolar membrane preparation conditions on the mechanical durability and electrochemical performance for electrodialysis applications

Allison M. Crow[1,2,3], Julia D. Lenef[3], Todd G. Deutsch[3], Wilson A. Smith[*1,2,3]

[1] Department of Chemical and Biological Engineering, University of Colorado Boulder, Boulder, Colorado 80303, United States

[2] Renewable and Sustainable Energy Institute, University of Colorado Boulder, Boulder, Colorado 80303, United States

[3] National Renewable Energy Laboratory, Golden, CO, USA 80401

[*]Corresponding author email - wilson.smith@colorado.edu

Catalyzed versus uncatalyzed force curves

T-peel force curves were measured at a pull rate of 10 mm/min for 50 mm for a total of 5 minutes of pull time. Examples of the resulting curves are shown below for the uncatalyzed (**Figure S1a**) and catalyzed (**Figure S1b**) tests. It can be seen that the uncatalyzed membranes show much more uniform force over the full pull distance as compared to the catalyzed samples. The noise of the catalyzed samples may be attributed to both the preparation methods as well as the presence of catalyst in the junction. Uncatalyzed samples were placed immediately from full hydration into the press for 2 minutes and then returned to hydration, leaving no time for drying.

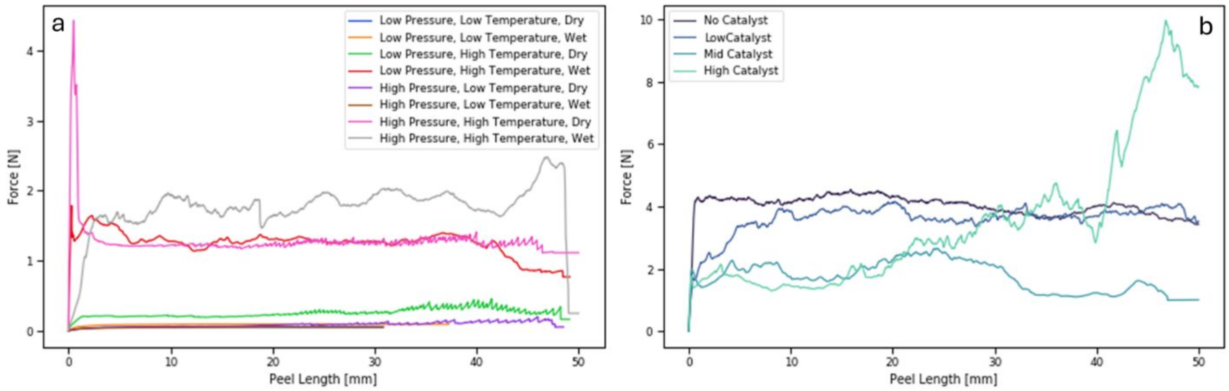

Figure S1. A comparison of the force curves produced when t-peeling uncatalyzed (a) and catalyzed (b) BPMs. The increase in catalyst increases the variability of the force measured throughout the t-peel experiment.

Catalyzing the membranes, however, required time to air spray the membranes which may lead to partial dehydration. This is particularly apparent when looking at the membrane that was air sprayed with DI water. If the preparation methods did not matter, those membranes should show very similar profiles to the uncatalyzed samples. Furthermore, it is hypothesized that the heterogeneity of the catalyzed membranes also contributes to the noise of their force curves since the water dissociation catalyst does not have the same mechanisms to form adhesive bonds as the monopolar membranes and the noise of the curve increases with increasing catalyst loading.

Because the catalyzed membranes were all made by presoaking in DI water and with a 100°C hot press, they all achieved a minimal level of adhesion: none of the catalyzed membranes delaminated immediately. This allowed these membranes to be stored in a dish of DI water whereas the membranes in the uncatalyzed study did not all meet this minimum level of adhesion and therefore were stored between soaked paper towels rather than free standing in water. This post press hydration also appears to increase the overall adhesion strength as force values for the catalyzed curves are generally higher than those for the uncatalyzed tests.

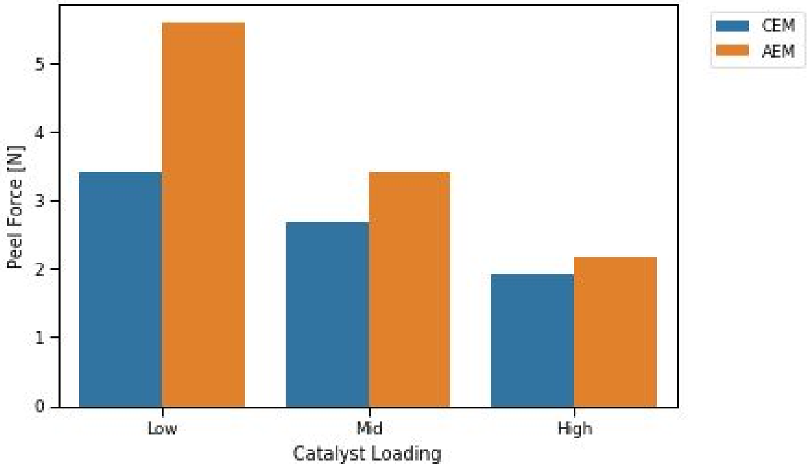

Figure S2. A comparison of t-peel adhesion strength dependent of whether the water dissociation catalyst was sprayed on to the AEM (orange) or CEM (blue). The adhesion force was measured higher for AEM application for all catalyst loadings investigated so catalyzed membranes for this study were fabricated by spraying catalyst on to the AEM and pressing on the CEM.

Determining the membrane for catalyst application

To determine whether to spray the TiO_2 water dissociation catalyst on the anion exchange membrane (AEM) or the cation exchange membrane (CEM), T-peel force measurements were compared between the two fabrication methods at three catalyst loadings. **Figure S2** shows that the BPMs fabricated by air spraying catalyst on to the AEM show consistently higher peel forces across all catalyst loadings. From these results it was determined that all membranes should be fabricated by applying catalyst to the AEM and then pressing the CEM onto the AEM and catalyst.

Additional SEM imaging and EDS after T-peel tests

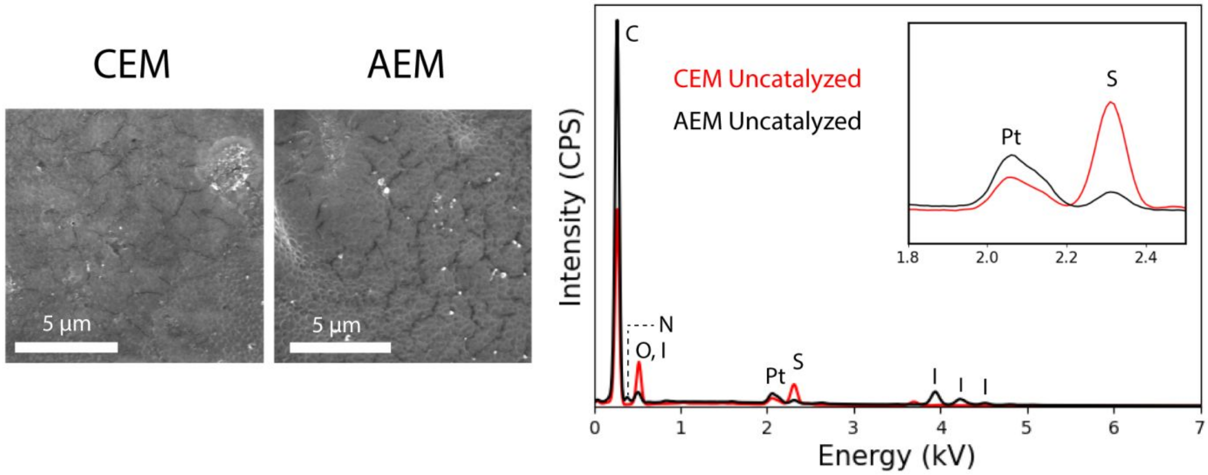

Figure S3. Uncatalyzed BPM at high pressure and high temperature after T-peel. SEM and EDS spectra of the CEM and AEM after T-peel. Sample is uncatalyzed with high pressure and high temperature hot press conditions. Visible texture on the surface of each membrane known as the "orange peel" effect is present as well as membrane cracking after drying.

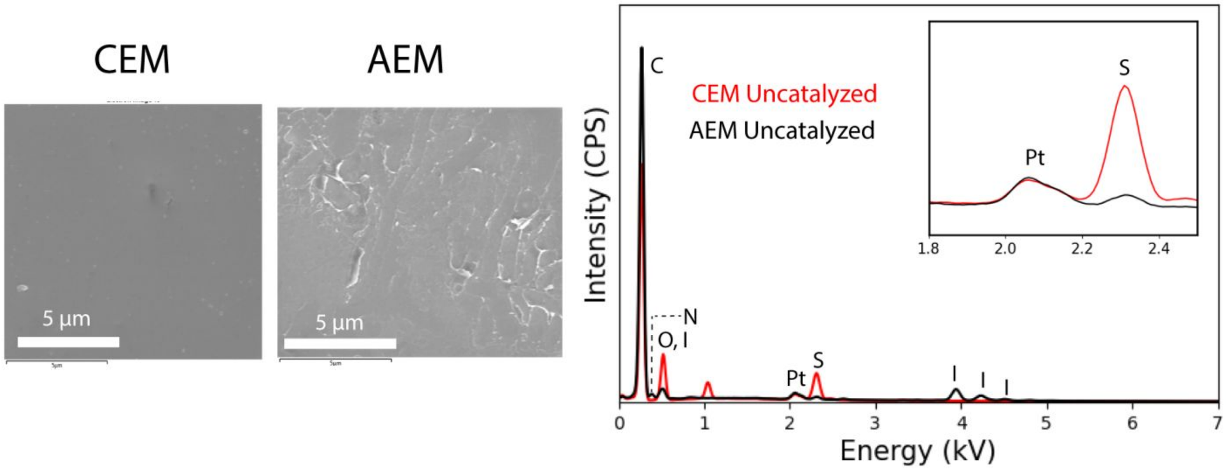

Figure S4. Uncatalyzed BPM at low pressure and high temperature after T-peel. SEM and EDS spectra of the CEM and AEM after T-peel. Sample is uncatalyzed with low pressure and high temperature hot press conditions. In general, both membranes surfaces are relatively smooth with some evidence of damage at the surface of the AEM.

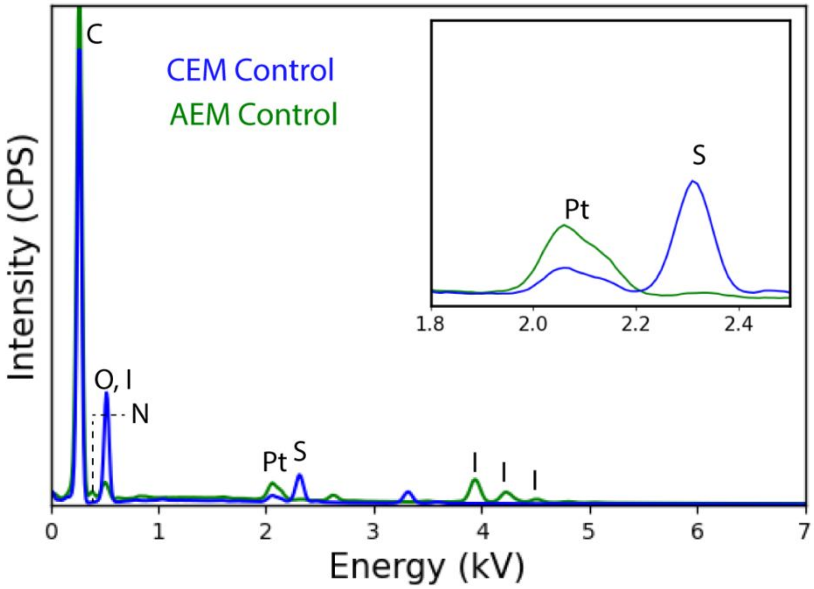

Figure S5. EDS spectra of AEM and CEM controls with no heat or pressure applied. No sulfur peak is present in the AEM control.
